# Label and quantify mRNA molecules in live cell experiments using SunRISER and dNEMO

**DOI:** 10.1016/j.xpro.2022.101630

**Published:** 2022-08-18

**Authors:** Yue Guo, Gabriel J. Kowalczyk, Robin E.C. Lee

**Affiliations:** 1 Department of Computational and Systems Biology, School of Medicine, University of Pittsburgh, Pittsburgh, PA 15260, USA; 2 Department of Physics and Astronomy, University of Pittsburgh, Pittsburgh, PA 15260, USA; 3 Center for Systems Immunology, School of Medicine, University of Pittsburgh, Pittsburgh, PA 15260, USA

**Keywords:** Bioinformatics, Microscopy, Molecular biology, Molecular/Chemical probes, Biotechnology and bioengineering

## Abstract

Visualization of mRNA molecules in single cells has revealed their core mechanisms as well as sources of cell-to-cell and spatiotemporal heterogeneity. Here, we describe a protocol to label, visualize, and quantify mRNA molecules by time-lapse imaging with the capability of resolving mRNA molecules over durations of hours to days. We provide links to mRNA-labeling plasmids as well as free software for a semi-automated image analysis pipeline.

For complete details on the use and execution of this protocol, please refer to Guo and Lee (2022) and Kowalczyk et al. (2021).

## Highlights

• SunRISER is a toolbox optimized for single mRNA live-cell imaging experiments
• Rapid detection of labeled single mRNA molecules in fluorescence microscopy images
• Automated single-cell segmentation and mRNA quantification

## Abstract (editor highlights)

Publisher’s note: Undertaking any experimental protocol requires adherence to local institutional guidelines for laboratory safety and ethics.

## Graphical abstract

**Figure:**
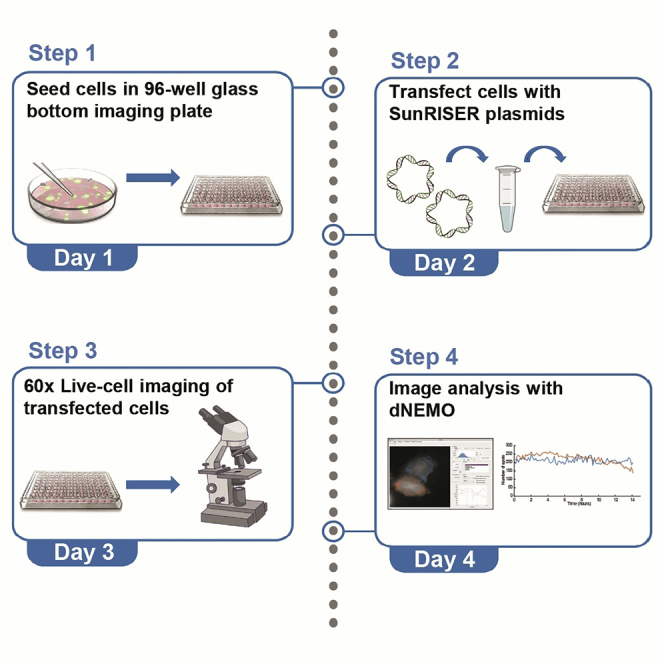

## Before you begin

The following protocol is for labeling and visualization of single mRNA molecules, followed by image analysis with a semi-automated pipeline. This protocol is described using HeLa cells but is adaptable to other mammalian cells.

### Label a gene of interest with stem-loops in mammalian cells

**Timing: 2 weeks**

**Figure 1:**
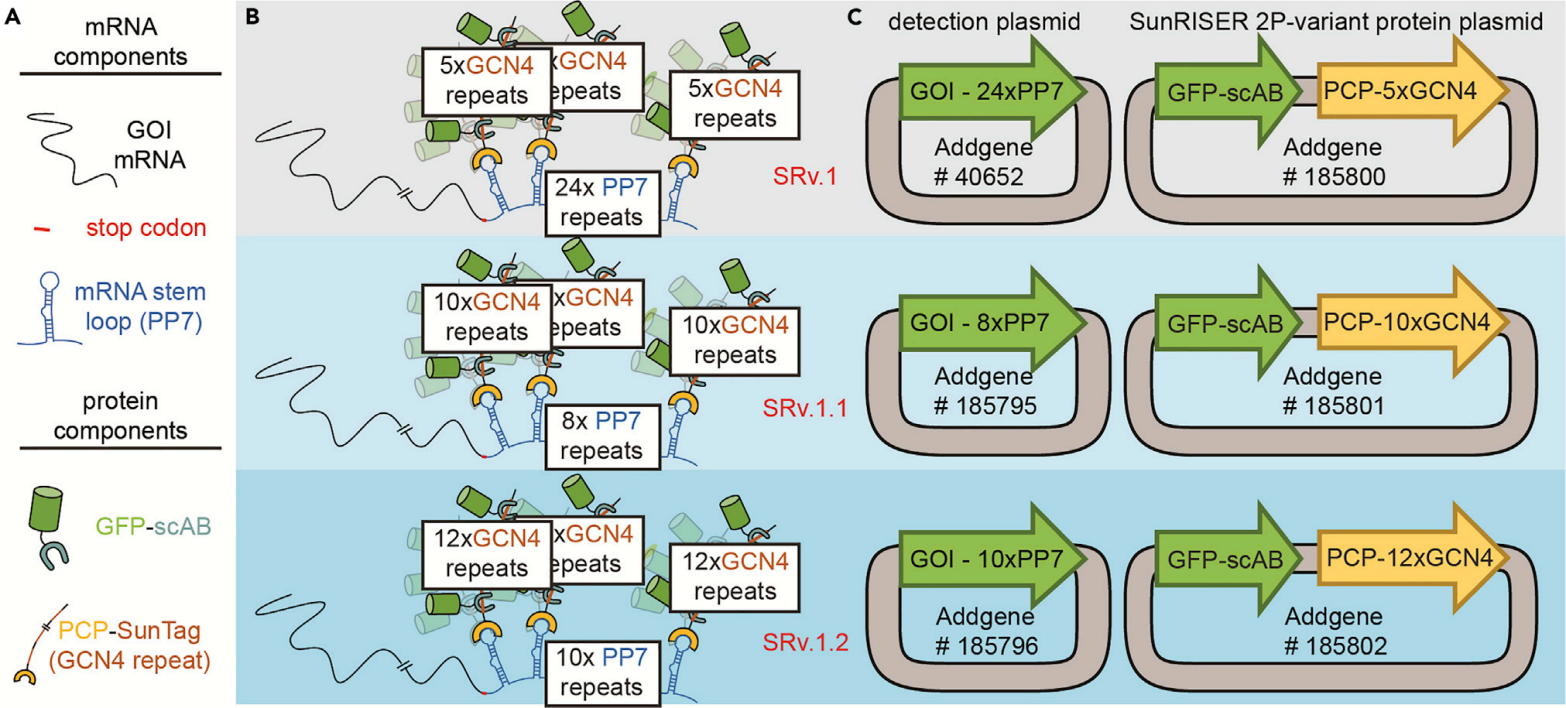
Schematic of components and plasmid combinations for typical SunRISER mRNA-labeling experiments (A) mRNA and protein components for SunRISER labeling experiments. (B) Schematics of SunRISER variants SRv.1 (top), SRv.1.1 (center), and SRv.1.2 (bottom) for fluorescence signal amplification. The mRNA transcript (black) is tagged at 3′ UTR with PP7 stem loops (blue). In the first stage of signal amplification, each stem loop can be bound by two PCP coat proteins (yellow) fused to a SunTag GCN4 peptide array (orange). In the second stage of signal amplification, GFP (green) is recruited through antibody-peptide-specific binding between scFv (gray) and GCN4 epitopes. (C) Plasmid maps of the SunRISER two-plasmid (2P) variants consisting of detection plasmids (left) and protein plasmids for SRv.1-2P (top), SRv.1.1-2P (center), and SRv.1.2-2P (bottom). Using the indicated plasmids from Addgene, the GOI CDS for SRv.1 (top, left) is CFP and the GOI CDS for SRv.1.1 and SRv.1.2 (center and bottom, left) is mCherry. Although the 2P variants of SunRISER are simpler to work with, SunRISER variants using three plasmids as described previously in (Guo and Lee, 2022) can be used as outlined previously and in Table 1.

Single-molecule imaging of mRNA has revealed fundamental properties of mechanisms in the central dogma that lead to variability between single cells. Continuous imaging of mRNA in living cells enables higher spatiotemporal resolution for mRNA shuttling and processing events that occur within the timescales of minutes, and more recently shown in the timescales of hours and days (Cawte et al., 2020; Guo and Lee, 2022; Tantale et al., 2016). To visualize single mRNA molecules for extended durations by wide-field microscopy, we developed a live-cell reporter called SunRISER (Guo and Lee, 2022). SunRISER uses a two-stage labeling approach (Figure 1). In the first stage, an mRNA of interest is extended in the 3′ UTR with a short array of bacteriophage-derived stem-loops (Bertrand et al., 1998; Chao et al., 2008). In the second stage of labeling, individual stem-loop structures are specifically bound by bacterial coat proteins (CP) tagged with a SunTag (Tanenbaum et al., 2014) array of epitopes (CP-SunTag). Fluorescence amplification on an mRNA occurs when CP-SunTag binds to a stem loop, and the SunTag epitope array recruits multiple scFv-GFP molecules (Figures 1A and 1B). Previously, we used computational modeling and experiments to establish the optimal SunRISER configuration. An important finding from this work was that a 5:1 protein expression ratio respectively for scFv-GFP and CP-SunTag is necessary for consistent imaging and detection of mRNA molecules. We subsequently demonstrated that a 5:1 ratio can be achieved in HeLa cells by transient transfection using CMV and UBC promoters to drive expression for fluorescent proteins, followed by microscopy and quantitative image analysis (Guo and Lee, 2022).

Although we have had success using this SunRISER configuration to label mRNAs in other human cells, such as A549, this may not always be the case for all cell lines. We therefore suggest for new cell lines, or for stable cell lines, to consider the SunRISER design as a starting point which may require further optimization at the level of promoters to achieve the optimal 5:1 protein expression ratio. We routinely evaluate expression of coding sequences (CDS) for fluorescent proteins for different promoters, using microscopy or flow cytometry to establish the average fluorescence among a population of cells.

Another important consideration is the choice of which RNA species to visualize for a SunRISER-labeling experiment. We evaluated SunRISER using the CDS for mCherry to represent a generic template that does not undergo particular regulation. In general, we expect that many SunRISER-labeling experiments will opt to switch the CDS template to other sequences (described below). See (Guo and Lee, 2022) for complete details on the modeling, optimization, and experimental validation of the SunRISER labeling system, as well as description of different SunRISER variants. Component plasmids for different versions of SunRISER (Figure 1C and Table 1) are available on Addgene (see the key resources table).

**Table 1:** SunRISER plasmid variants

SunRISER system	Detection plasmid	pcp-nxSunTag	scFv-GFP
Variation 1 – SRv.1	Phage-cmv-CFP-24×pp7 (Addgene #40652)	ubc-nls-pcp-5×SunTag (Addgene #185797)	cmv-sfgfp-gb1-scAB (Addgene #185794)
Variation 2 – SRv.1-2P	Phage-cmv-CFP-24×pp7 (Addgene #40652)	cmv-sfgfp-gb1-scAB-ubc-nls-pcp-5×SunTag (Addgene #185800)	
Variation 3 – SRv.1.1	cmv-mCherry-8×PP7 (Addgene #185795)	ubc-nls-pcp-10×SunTag (Addgene #185798)	cmv-sfgfp-gb1-scAB (Addgene #185794)
Variation 4 – SRv.1.1-2P	cmv-mCherry-8×PP7 (Addgene #185795)	cmv-sfgfp-gb1-scAB-ubc-nls-pcp-10×SunTag (Addgene #185801)	
Variation 5 -SRv.1.2	cmv-mCherry-10×PP7 (Addgene #185796)	ubc-nls-pcp-12×SunTag (Addgene #185799)	cmv-sfgfp-gb1-scAB (Addgene #185794)
Variation 6 -SRv.1.2-2P	cmv-mCherry-10×PP7 (Addgene #185796)	cmv-sfgfp-gb1-scAB-ubc-nls-pcp-12×SunTag (Addgene #185802)	

Clone the gene of interest (GOI) into a reporter expression vector suitable for the specific application. Among the SunRISER plasmid toolkit, we provide 2 different stem-loop array lengths of PP7 stem-loops (8×PP7, 10×PP7), and plasmids for 24×PP7 are also available (Addgene #40652). In these plasmids, the CDS for the mCherry reporter gene is flanked by standard restriction enzymes to facilitate replacement with another GOI via typical molecular cloning methods. Although the plasmids have repeat sequences which can lead to technical difficulties, in our experience we have had no issues inserting GOIs and plasmid amplification in typical *E. coli* strains such as DH5-alpha is routinely successful. See (Guo and Lee, 2022) or Addgene for further information and plasmid maps. The resulting plasmid containing the GOI with stem loop extensions is referred to as the ‘detection plasmid’.

## Key resources table

**Table:** 

REAGENT or RESOURCE	SOURCE	IDENTIFIER
**Chemicals, peptides, and recombinant proteins**		

Fugene HD	Promega	Cat # E2311
Opti-MEM, Reduced Serum Medium	Thermo Fisher Scientific	Cat # 31985062
DMEM	Corning	Cat # 10-017-CV
FBS	Corning	Cat # 35-010-CV
Penicillin-Streptomycin (10,000 U/mL)	Thermo Fisher Scientific	Cat # 15140122
L-Glutamine (200 mM)	Thermo Fisher Scientific	Cat # 25030081
FluoroBrite™ DMEM	Thermo Fisher Scientific	Cat # A1896701

**Experimental models: Cell lines**		

Human: HeLa cell	ATCC	RRID: CVCL_0030

**Recombinant DNA**		

cmv-sfgfp-gb1-scAB	(Guo and Lee, 2022)	Addgene #185794
cmv-mCherry-8×PP7 (detection plasmid)	(Guo and Lee, 2022)	Addgene #185795
cmv-mCherry-10×PP7 (detection plasmid)	(Guo and Lee, 2022)	Addgene #185796
ubc-nls-pcp-5×SunTag (SRv.1)	(Guo and Lee, 2022)	Addgene #185797
ubc-nls-pcp-10×SunTag (SRv.1.1)	(Guo and Lee, 2022)	Addgene #185798
ubc-nls-pcp-12×SunTag (SRv.1.2)	(Guo and Lee, 2022)	Addgene #185799
cmv-sfgfp-gb1-scAB-ubc-nls-pcp-5×SunTag (SRv.1-2P)	(Guo and Lee, 2022)	Addgene #185800
cmv-sfgfp-gb1-scAB-ubc-nls-pcp-10×SunTag (SRv.1.1-2P)	(Guo and Lee, 2022)	Addgene #185801
cmv-sfgfp-gb1-scAB-ubc-nls-pcp-12×SunTag (SRv.1.2-2P)	(Guo and Lee, 2022)	Addgene #185802
phage-cmv-cfp-24×pp7 (detection plasmid)	(Wu et al., 2012)	Addgene #40652
5-alpha Competent *E. coli* (high efficiency)	New England Biolabs (NEB)	Cat # C2987H

**Software and algorithms**		

ImageJ	(Schneider et al., 2012)	https://imagej.nih.gov/ij/
dNEMO	(Kowalczyk et al., 2021)	Database: https://github.com/recleelab, https://doi.org/10.5281/zenodo.6841307
Cellpose	(Stringer et al., 2021)	Database: https://github.com/MouseLand/cellpose
Python	Python Software Foundation	https://www.python.org
Digital resources associated with this protocol	This paper	https://doi.org/10.17632/8j4x6dj2f7.1

**Other**		

DeltaVision Elite	GE	N/A
96-well glass bottom plate	MatriPlate	Cat # MGB096-1-2-LG-L

## Step-by-step method details

### SunRISER plasmids delivery into HeLa cells to label single mRNAs

**Timing: 4 days**

This step allows delivery of GOI labeled with stem-loops and SunRISER labeling component plasmids via transient transfection.

1. Choose the appropriate SunRISER setup for your application.
  a. Choose the variant of SunRISER that favors either a shorter or longer stem-loop extension (Figure 1 and Table 1) and select the appropriate detection plasmid available from Addgene (SRv.1: 24×PP7; SRv.1.1: 8×PP7; SRv.1.2: 10×PP7) or use the user-generated detection plasmid as described in the ‘before you begin’ section above.
    ***Note:*** Although SRv.1.1 and SRv.1.2 extend the target mRNA with a smaller stem-loop array than SRv.1, the associated protein component (consisting of pcp-nxSunTag and scFv-GFP proteins, see Figure 1) of the labeled-mRNA complexes for SRv.1.1 and SRv.1.2 are larger than for SRv.1. Because of this complementarity, SRv.1 and SRv.1.2 are similar in overall molecular weight. The overall molecular weight of labeled mRNA complexes for SRv.1.1 is approximately 35% smaller than for SRv.1 and SRv.1.2, as described in (Guo and Lee, 2022). For all SunRISER variants, the mRNA:protein composition of the labeled mRNA is balanced for optimal signal-to-background. The user can therefore select a SunRISER variant that works best with their application. For example, extending shorter mRNAs with a smaller stem-loop array reduces sequence perturbations, which may be favorable, but may not necessarily lead to the best mRNA detection. The choice of SunRISER variant should ultimately weigh the particular application and experimental goals against empirical imaging results.
  b. Based on the stem-loop lengths, choose the corresponding SunTag array lengths. (SRv.1: 24×PP7-5×SunTag; SRv.1.1: 8×PP7-10×SunTag; SR.v1.2: 10×PP7-12×SunTag).
  c. Choose the plasmid version suitable for your system.
    ***Note:*** In SunRISER, we provide both a single plasmid encoding both scFv-GFP and pcp-nxSunTag and a version where the two constructs are on separate plasmids (see Figure 1 and Table 1). Separate plasmids can be useful in determining proper expression ratios are achieved as well as to alleviate any unexpected complications that could arise from a double-expression plasmid.
  d. Calculate the amount of each component based on the selected version of SunRISER.
    **CRITICAL:** For three-plasmids, equal molar amounts of each plasmid (1:1:1) is optimal. Since the three plasmids as supplied have comparable size, this also works out to a 1:1:1 weight ratio. Similarly, a 1:1 molar ratio is optimal for the two-plasmid version. For the 2-plasmid version as supplied, note that a 1:1 molar ratio equates to a 1.5:1 weight ratio because the double expression plasmid as supplied is approximately 1.5× the size of the detection plasmid. This ratio may change depending on the size of the GOI.
2. Seed HeLa cells in 96-well imaging plates.
  a. Seed 1 × 10^4^ HeLa cells per well of 96-well glass bottom imaging plates (Matriplate; .17 mm Flat Clear Glass Bottom) in 300 μL of DMEM (supplemented with 10% FBS, 1% streptomycin/penicillin, and 1% L-glutamine).
    ***Note:*** While culture conditions for HeLa cells as described in this protocol do not require coating (fibronectin, collagen, etc.), other cell lines may. We do not expect any compatibility issues in imaging SunRISER with cell lines using fibronectin, poly-L and poly-D lysine. Other coatings such as collagen should be tested to ensure they do not have autofluorescence properties that interfere with fluorescence imaging.
  b. Incubate cells at 37°C in 5% CO_2_ incubator overnight (18–24 h) to allow cells to recover and adhere to the plate at approximately 70% confluency.
3. Transfect cells with SunRISER plasmids using FuGENE® HD following manufacturer’s instructions.
  a. Warm FuGENE® HD Transfection Reagent to 22°C.
  b. Mix DNA and FuGENE® HD Transfection Reagent according to manufacturer’s instructions. For this protocol, we mixed 150 ng total plasmid with 0.45 μL FuGENE® HD Transfection Reagent in 10 μL Opti-MEM™ for each well. [See troubleshooting Problem 1]
  c. Incubate the mixture for 10–15 min at 22°C.
  d. Add mixture into each well and mix by pipetting.
  e. Return cells to incubators for 24–48 h. Post-transfection media is supplemented with 10% FBS, 1% streptomycin/penicillin, and 1% L-glutamine. The cells should be approximately 85% confluent post-transfection.

***Note:*** Each step can be optimized with the instructions from the FuGENE® HD transfection protocol. Although we have only used FuGENE® HD for our SunRISER-labeling experiments, we expect other transfection reagents and techniques (such as electroporation) will lead to equivalent results.

### Imaging of SunRISER-labeled mRNAs

**Timing: 2 days**

**Figure 2:**
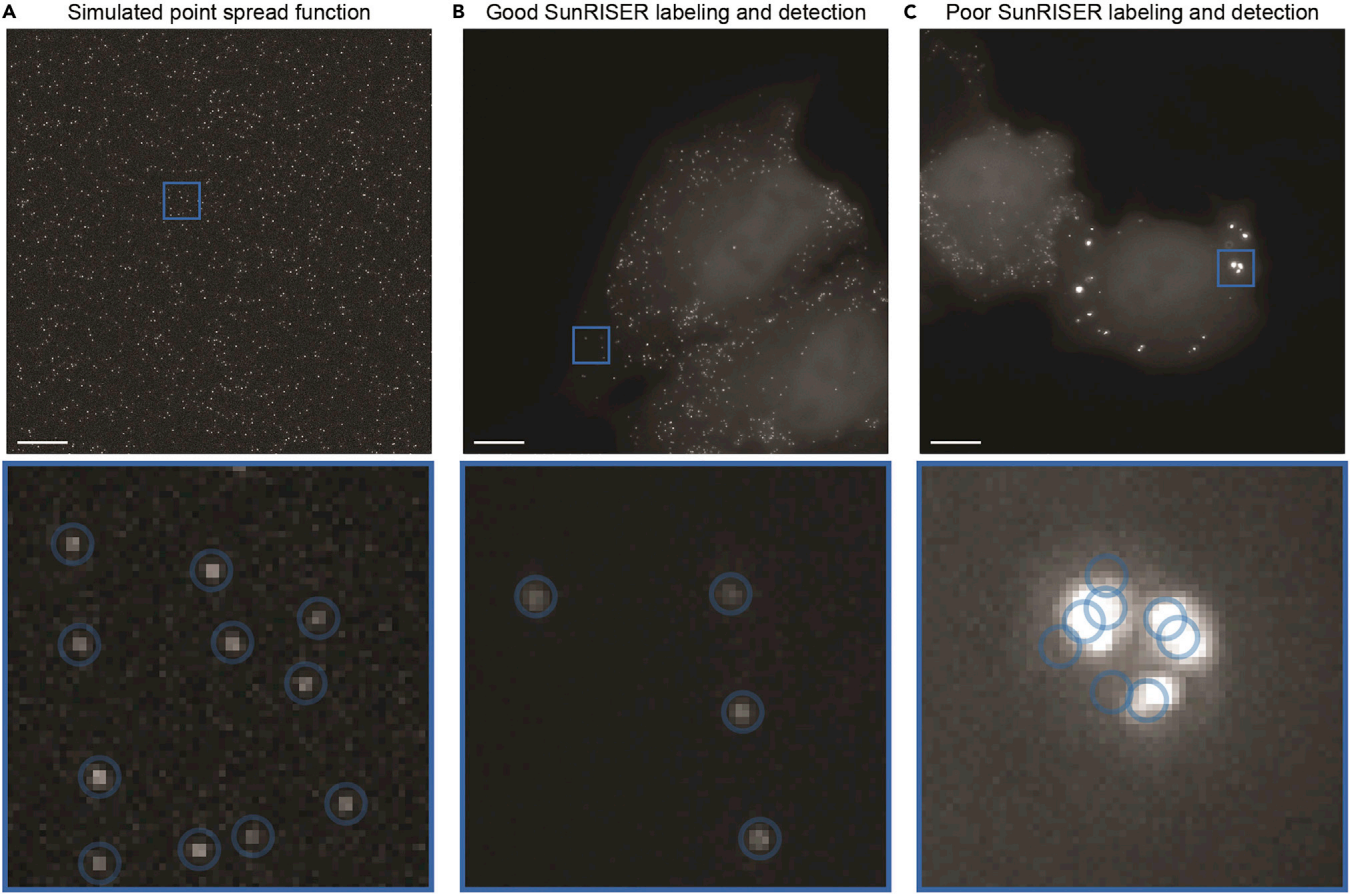
SunRISER-labeled mRNAs appear as diffraction-limited spots (A) Image of theoretical point spread functions (PSFs) for diffraction limited signals. The simulated image was generated as described previously (see (Kowalczyk et al., 2021)). (B and C) Representative maximum intensity projections of HeLa cells transfected with SunRISER SRv.1-2P and detection plasmid CFP-24×PP7 (top images). Bottom images represent detail of fluorescence images as indicated, with blue circles representing spots detected by dNEMO analysis. In good labeling conditions, (B) mRNA molecules labeled with SunRISER appear as diffraction-limited spots comparable to those generated in the simulated image. In poor labeling (C) fluorescent structures are larger than diffraction-limited objects and do not represent single mRNA molecules. Large objects also show evidence of oversegmentation where a single fluorescent structure is detected as multiple spots. Scale bars: 10 μm.

This step allows imaging of SunRISER-labeled mRNAs for up to 24 h. For time-lapse live cell imaging we used a DeltaVision Elite microscope (GE Healthcare) equipped with a 60× NA1.42 oil-immersion objective, sCMOS camera, solid-state illumination module, and an environmental controlled chamber (37°C, 5% CO_2_). A minimum of 60× magnification is required to resolve single mRNA puncta as diffraction-limited objects (Figure 2, see also (Guo and Lee, 2022)).

***Note:*** We expect any epifluorescence microscope equipped with an environment control chamber can be used for imaging SunRISER-labeled mRNAs. Other more advanced microscopes (e.g., confocal, light sheet, and many others) are likely to produce SunRISER images with even greater signal-to-background. We have successfully imaged SunRISER in 24-hour experiments and expect that longer duration experiments may also be possible.

4. Before beginning, replace complete growth medium with FluoroBrite medium (supplemented with 10% FBS, 1% streptomycin/penicillin, and 1% L-glutamine).

***Note:*** FluoroBrite or any phenol-red free cell culture medium is recommended to increase the signal-to-background of fluorescence images.

5. Place the imaging plate in the microscope incubation chamber to allow the whole system to equilibrate for at least 30 min before the experiment starts.
6. Set up appropriate imaging conditions for live cell imaging. For 24-h imaging, we select FITC as Ex and Em filters and set the exposure time to 0.005 s and ND filter to 10%. Images were taken as Z-stacks with 1 μm slices spacing and total thickness 5 μm every 10 min for 24 h. Data acquisition was performed in 1024 × 1024-pixel format.

***Note:*** Multi-channel imaging using spectrally compatible filter sets can be used to select for successfully transfected cells based on expression of mCherry or CFP if these are the GOIs expressed from the detection plasmid.

***Note:*** For a typical widefield microscope equipped with a NA1.4 60× oil objective, 0.3 μm z-spacing will be approximately the ideal Nyquist sampling, where each diffraction-limited object will appear in at least two to three consecutive z-slices. Although the z-spacings between 0.5 and 1 μm will not oversample as effectively as Nyquist conditions, it allows coverage over wider axial range with fewer exposures at each time point. In our experience these settings lead to quantitatively similar results for mRNA numbers, while reducing the effects of photobleaching and phototoxicity in long-term experiments. For particular applications, ideal combinations of z-spacing and z-slice numbers can be chosen to favor oversampling for increased spatial resolution in the z-axis, or increased depth, or reduced molecular and cellular strain as appropriate to the usage case. Experiments demonstrating resistance to photobleaching with these settings can be found in (Guo and Lee, 2022).

7. Select cells expressing SunRISER-labeled mRNA as diffraction-limited spots and mark the region of interest for live-cell imaging experiments.

***Note:*** Imaging conditions are considerably variable between different microscope setups. SunRISER-labeled mRNAs appear as diffraction-limited spots (Figures 2A and 2B), and the raw pixel intensity range for the spots detected under the imaging conditions described in this protocol have an average intensity of 673 +/- 189 units from a 16-bit sCMOS detector when pixel values are examined in image analysis software like ImageJ (Schneider et al., 2012). For cells expressing lower scFv-GFP abundance, mRNA spots can be difficult to distinguish with a poor signal-to-background ratio and can suffer from rapid photobleaching if the excitation illumination is too intense. [See troubleshooting Problem 2]

**CRITICAL:** Transient transfection can lead to subpopulations of cells with GFP expression that is significantly higher than average. We typically avoid imaging and analysis of cells expressing too much GFP that show very bright background fluorescent intensity, and cells with spots that are larger than the diffraction limit Figure 2C). [See troubleshooting Problem 3]

8. When possible, use Ultimate Focus or a related auto-focusing mechanism to prevent the loss of the imaging planes during long-term imaging experiments. [See troubleshooting Problem 4]
9. Begin time-lapse imaging experiment.

**Figure 3:**
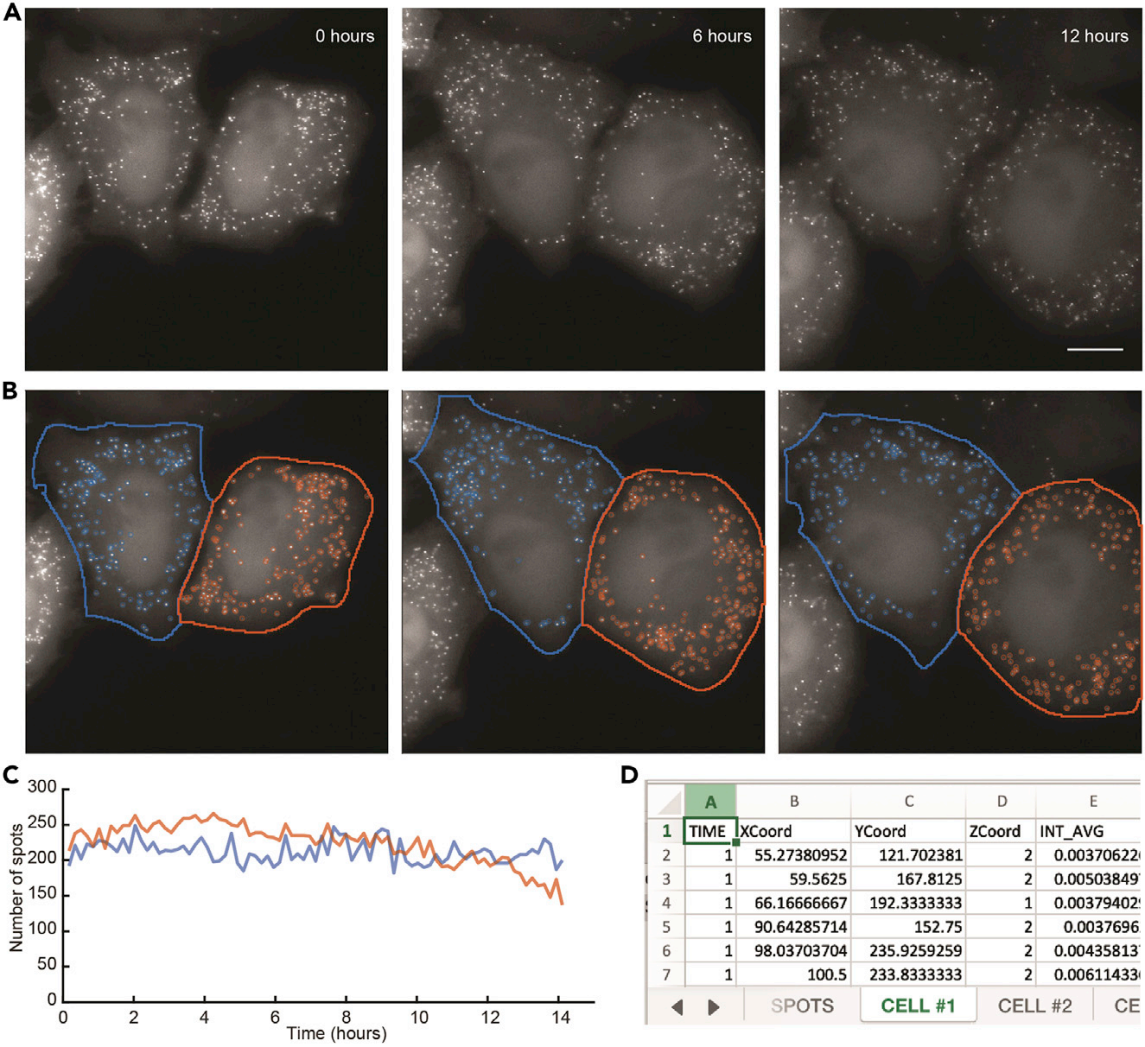
Quantification of SunRISER-labeled mRNAs in time-lapse live-cell images with dNEMO (A) Maximum intensity projection of HeLa cells transfected with SunRISER SRv.1-2P and detection plasmid CFP-24×PP7. Cells were imaged every 10 min for 24 h. Scale bar: 10 μm. (B) SunRISER-labeled mRNAs detected by dNEMO (blue or orange circles) and associated with cells segmented using Cellpose. (C) Time-courses for the number of mRNA molecules identified within the 2 cells shown in (A). (D) Example results file generated by dNEMO and output to excel. Features are collected for every spot, and spots within each single cell are separated by tabs.

***Note:*** The resulting movie file can be opened with ImageJ and quantified with dNEMO as described below. The included file ‘SunRISER_SAMPLE_MOV.tif’ (https://doi.org/10.17632/8j4x6dj2f7.1; see also, Figure 3A) is representative of a typical imaging experiment result using HeLa cells transfected with stock 24×PP7 and SRv.1-2P plasmids as described here.

### Image analysis with dNEMO and Cellpose

**Timing: ∼20 min (for sample SunRISER movie with 145 time points each with 4 z-slices)**

***Note:*** The timing for this step can increase depending on the number of timepoints in a given time-lapse series, the number of cells in the given image, and the hardware running both dNEMO and Cellpose. The reported time is given for the sample SunRISER image provided alongside the software on a 2015 MacBook Pro laptop (16 GB RAM, 2.5 GHz processor).

This section describes the semi-automated image analysis which identifies individual mRNA transcripts, extracts the fluorescence intensity from each transcript, and assigns mRNAs to individual cells to create single-molecule single-cell datasets over time-lapse images (Figure 3). We use the spot detection and quantification tool dNEMO (detecting-NEMO) which is optimized for rapid and accurate detection of punctate structures (spots) in time-lapse fluorescence microscopy images. While dNEMO contains dedicated tools for the manual segmentation of individual cells, the most recent version of the software interfaces with Cellpose (Stringer et al., 2021), a generalist algorithm for automated cell and nucleus segmentation. Cellpose automatically segments multiple cells in each input image and typically performs better when cells are sub-confluent. With Hela cells, we typically have less than 5 cells per 60× image with a 1024 × 1024 sCMOS detector. Individual cells are reconstituted as single-cell trajectories when input into dNEMO. In this protocol, the combination of dNEMO and Cellpose is used for automated spot detection, cell segmentation, and cell tracking. Sample images acquired using SunRISER and the corresponding results files from both dNEMO and Cellpose are provided with this protocol (https://doi.org/10.17632/8j4x6dj2f7.1). Also included for users is an additional sample image of cells labeled with smFISH probes and corresponding dNEMO/Cellpose results files.

***Note:*** There are standalone executables of the dNEMO software available with this protocol (https://doi.org/10.17632/8j4x6dj2f7.1) which do not require MATLAB to be installed. Use of Cellpose and dNEMO outside of the standalone executables requires installation of Python 3, MATLAB, and the OME bioformats package. Detailed installation instructions can be found in the dNEMO documentation on Database: https://github.com/recleelab, https://doi.org/10.5281/zenodo.6841307.

***Optional:*** If Cellpose is not installed, dNEMO implements a manual cell segmentation tool which can be used to segment cells within the dNEMO interface.

**Figure 4:**
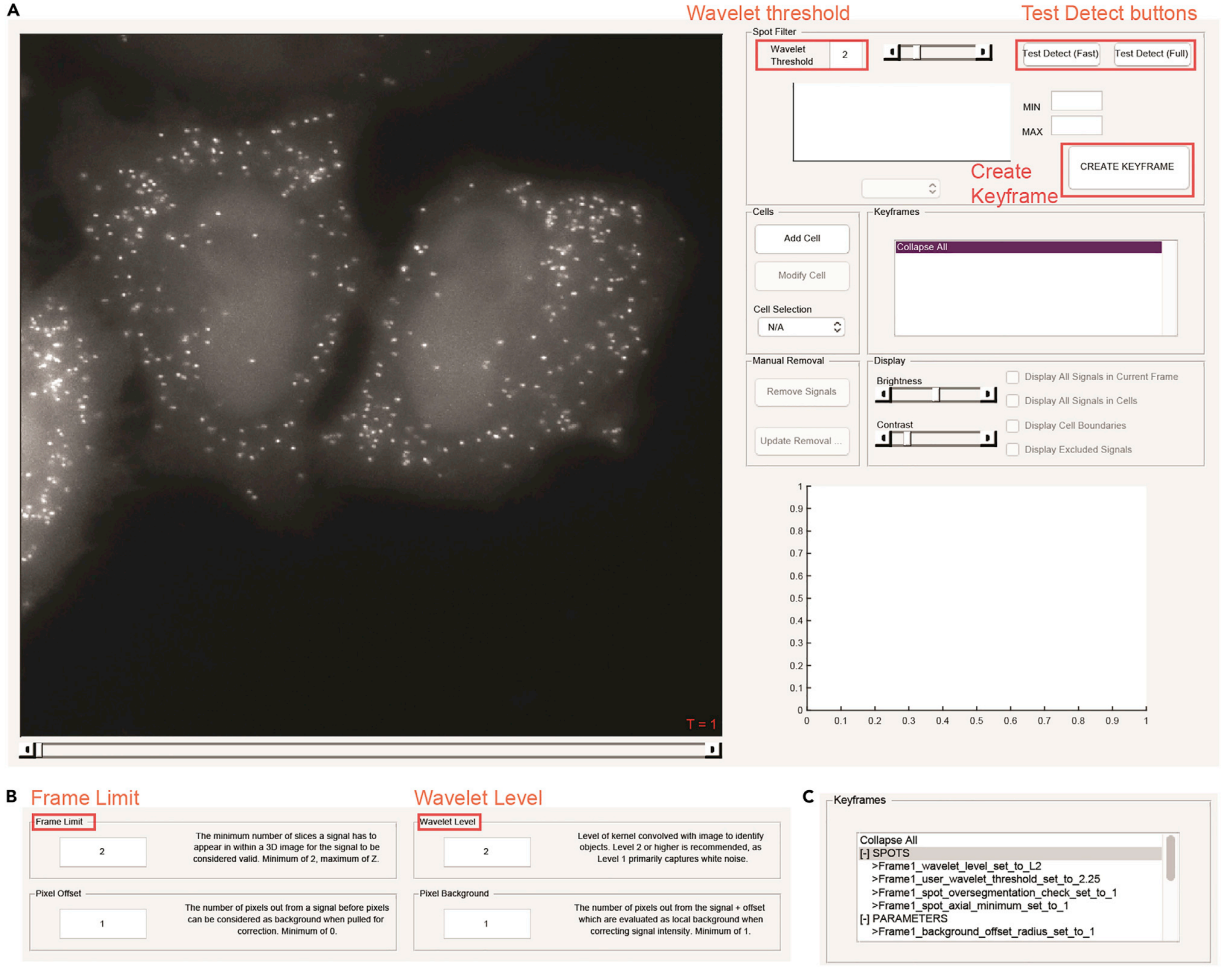
Spot detection settings and operations in dNEMO (A) The dNEMO software interface with an open image of HeLa cells transfected with SunRISER SRv.1-2P and detection plasmid CFP-24×PP7. Highlighted within the ‘Spot Filter’ panel (upper-right) are critical user operations: the ‘Wavelet Threshold’ value; the detection of spots over the currently displayed image (‘Test Detect’ buttons); and the generation of a keyframe of the current detection settings (‘Create Keyframe’ button). (B) Settings GUI accessible in dNEMO which holds user-defined parameters for spot detection. Highlighted are the ‘Frame Limit’ parameter (upper left) and the ‘Wavelet Level’ parameter (upper right). (C) Screenshot of the ‘Keyframes’ panel after the spot detection operation has completed operating over the time-lapse images.

**Figure 5:**
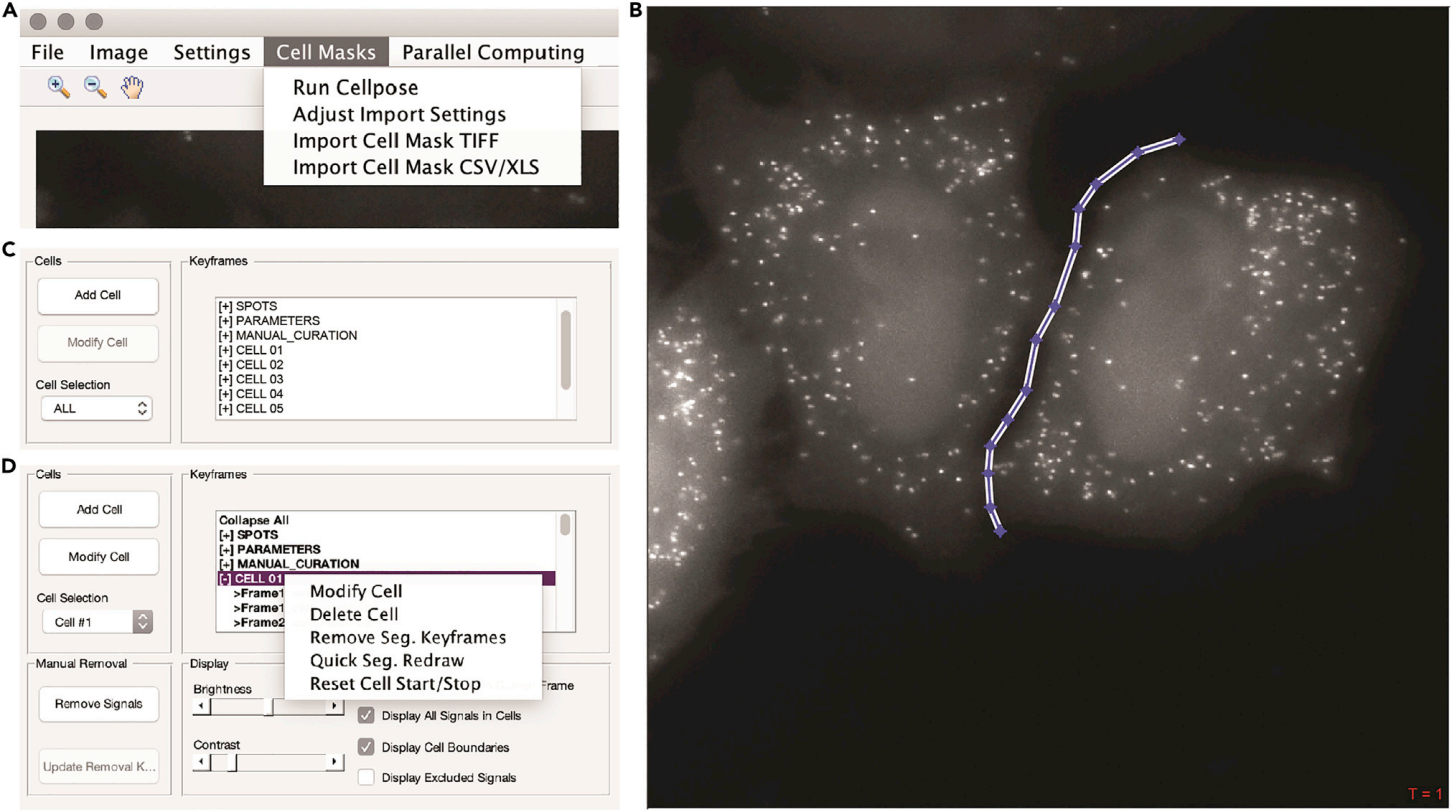
dNEMO interfaces with Cellpose for automated segmentation of cells (A) Screenshot of ‘Cell Masks’ drop-down menu to access Cellpose for cell segmentation over the currently displayed image as well as importing previously generated cell segmentation mask files into dNEMO. (B) Manual segmentation function operating over a displayed image in dNEMO with a user-defined polygon. (C) Screenshot of the ‘Cells’ and ‘Keyframes’ panels after Cellpose has completed segmentation and imported the resulting masks into dNEMO. (D) Screenshot of the user operations available to edit imported Cellpose results within the dNEMO interface after right clicking a cell within the ‘Keyframes’ panel.

**Figure 6:**
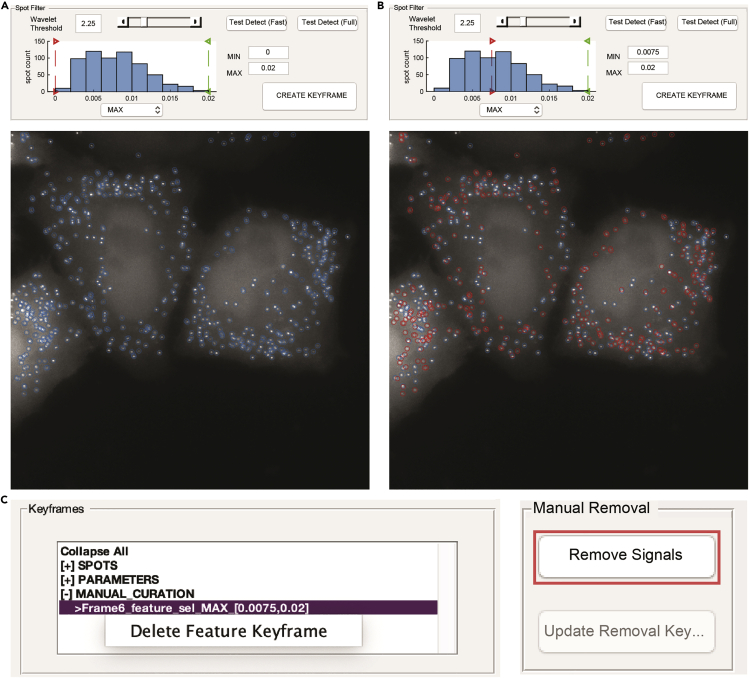
Keyframing and manual exclusion tools for curation of spot datasets (A and B) Screenshots of the feature selection in dNEMO for user-assisted filtering of spots based on their size or intensity, among other features. User-defined parameters for the maximum intensity of detected spots set with a lower bound of 0 (A, top) or 0.0075 (B, top). Resulting dNEMO spots either accepted (blue) or filtered (red) based on analysis with the values as shown (bottom). (C) Keyframe information window after clicking the ‘Create Keyframe’ button using the intensity-filtering settings in B (Left). The results of the filter can be deleted by clicking the entry. The ‘Manual Removal’ panel (Right) with the spot removal operation indicated (highlighted, red) is used to supplement user-assisted filtering, enabling keyframe entries for removal of user-selected spots from further analysis.

10. Open the time-lapse image in dNEMO.
  a. To open the standalone dNEMO executable, navigate to the dNEMO application within the executable folder and double-click the application icon.
    ***Alternatives:*** If using the MATLAB script package, type the following into MATLAB’s command window:
    >addpath(fullfile(cd, dNEMO_MATLAB_scripts))
    >RUN_ME
  b. With the interface open, navigate to File > Load Images. Select the image to be analyzed in the subsequent file selection pop-up window. The image ‘SunRISER_SAMPLE_MOV.tif’ is provided with the software for this protocol (https://doi.org/10.17632/8j4x6dj2f7.1) and can be opened and analyzed using the settings and steps described here. This image (Figures 3 and 4A) is also depicted in subsequent figures as an example for detection of SunRISER-labeled mRNAs within the dNEMO interface.
    ***Note:*** Image formats supported by BioFormats (Linkert et al., 2010) can be opened by dNEMO. Upper limit on image size may vary with available system memory.
11. Run spot detection on the currently displayed image.
  a. Select ‘Test Detect (Full)’ in the upper right ‘Spot Filter’ panel. This will run the spot detection algorithm over the currently displayed image (Figure 4A).
  b. Confirm visually that all spots are being detected by toggling the ‘Display All Signals in Current Frame’ toggle in the ‘Display’ panel.
    i. Adjust the value in the ‘Wavelet Threshold’ box (Figure 4A) and click ‘Test Detect (Full)’ (Figure 4A), both in the ‘Spot Filter’ panel, to re-run spot detection with an updated threshold value. The higher this value is, the fewer objects will be considered above the watershed threshold and thus detected. For images shown detecting SunRISER-labeled mRNAs in this protocol, the ‘Wavelet Threshold’ value was set to 2.25.
      **CRITICAL:** Properly detecting spots in an image requires examining the detected spots and adjusting the parameters as needed. Two additional parameters for spot detection that affect the generation of the wavelet map and cross-referencing the resulting wavelet maps of a 3D image stack can be found in Settings > Signal Parameters (Figure 4B). The ‘Frame Limit’ parameter indicates the number of consecutive slices in a 3D stack a spot must be detected in to be considered valid. In order to detect spots which only appear in one slice, for example, this value would need to be set to 1. The ‘Wavelet Level’ parameter dictates the level of the wavelet transform to use when generating the wavelet map to detect spots. The higher this value is, the larger the objects detected will be in the resulting wavelet map. In detecting the SunRISER-labeled mRNAs for this protocol, the ‘Wavelet Level’ was set to 2 and the ‘Frame Limit’ was set to 1. All other settings were kept at the default values. For complete details on the implementation of the wavelet transform and watershed segmentation operations in dNEMO, see (Kowalczyk et al., 2021). [See troubleshooting Problem 5]
  c. Once satisfied that the spots are being accurately detected, run spot detection with the current detection parameters over all images in the time-series by clicking ‘Create Keyframe’ in the upper right ‘Spot Filter’ panel (Figure 4A). The Keyframes box (Figure 4C) will update with information for detected spots and the associated parameters.
12. Run Cellpose to segment single cells over the time-lapse image sequence.
  a. Initiate Cellpose from dNEMO by navigating to Cell Masks > Run Cellpose (Figure 5A). This will startup Cellpose and run on the movie currently loaded into dNEMO. [See troubleshooting Problem 6]
  b. Upon completion, dNEMO will display a prompt for confirming the imported mask. Click ‘Ok’ to confirm the mask import.
    ***Note:*** Cellpose does not provide any tracking of the segmented cells. Cells are tracked over time using several parameters found in dNEMO by navigating to Cell Masks > Adjust Import Settings. For more information on how these parameters function see documentation of dNEMO at Database: https://github.com/recleelab, https://doi.org/10.5281/zenodo.6841307.
    ***Alternatives:*** If Cellpose is not installed on your system, manual segmentation is an option implemented in dNEMO. Similarly, cell masks generated in other applications can be imported using the ‘Cell Masks’ dropdown menu provided the masks are in a compatible matrix format (TIFF or excel/csv spreadsheet). Manual segmentation uses a process called keyframing where user-defined cell boundaries are propagated across frames of a time-lapse image. Briefly, the ‘Add Cell’ button of the ‘Cells’ panel will create an interactive polygon drawing tool over the current image (Figure 5B). Clicking on the image will begin the manual segmentation process and completing the polygon will create a new cell. The polygon for each cell can be left alone or modified as needed to create subsequent keyframes to refine changes to cell morphology or position at later time points. See (Kowalczyk et al., 2021) for full description of keyframing for cell segmentation and additional spot detection parameters.
  c. Cells can be adjusted using both the ‘Cells’ and ‘Keyframes’ panels in dNEMO.
    i. The slider along the bottom of the image can be used to navigate through the frames of a time-lapse image.
    ii. Select a cell using the ‘Cell Selection’ dropdown menu in the ‘Cells’ panel or clicking on a cell in the ‘Keyframes’ panel (Figure 5C).
    iii. Segmentations can be adjusted for the current frame by clicking the ‘Modify Cell’ button in the ‘Cells’ panel.
    iv. Multiple operations to adjust segmentations over time and deleting a cell can be accessed by right-clicking on a cell in the ‘Keyframes’ panel and selecting an option from the pop-up menu (Figure 5D). In conjunction these can be used to modify segmentations or delete inaccurate segmentations. For complete details on dNEMO’s keyframing functions, see (Kowalczyk et al., 2021) and Database: https://github.com/recleelab, https://doi.org/10.5281/zenodo.6841307. [See troubleshooting Problem 7]
13. Further curate data using keyframing tools and manual exclusion tools.
  a. Spots can be curated in an automated fashion by assigning keyframes for detected spots’ physical features. For example, using the histogram display axis in the ‘Spot Filter’, detected transcripts can be limited to those which have maximum intensities greater than or equal to 0.0075 by selecting ‘Max’ from the dropdown menu below the axis and typing 0.0075 into the ‘Min’ value to the right of the axis (Figures 6A and 6B).
  b. Click ‘Create Keyframe’ in the spot filter panel to create a new keyframe for this feature.
    ***Note:*** This creates a parameter for the upper and lower bounds of fluorescence intensity for spots deemed as acceptable. These bounds are propagated over the entire movie and can be adjusted by creating additional keyframes at different time-points. The bounds can also be deleted by selecting and right-clicking a given parameter in the ‘Manual Curation’ section of the ‘Keyframes’ panel (Figure 6C).
  c. Detected spots which are determined erroneous (e.g., lysosomal accumulation of fluorescent molecules) or oversegmented (Figure 3C) can be curated manually using the manual removal tool. [See troubleshooting Problem 8]
    i. Navigate to the ‘Manual Removal’ panel and click ‘Remove Signals’ (Figure 6C).
    ii. A crosshair will replace the mouse pointer icon when hovering over the main image axis in dNEMO. Clicking on detected spots in the image will remove them. Clicking on them again will undo the removal. Clicking and dragging the cursor will create a box allowing removal of numerous detected spots.
    iii. Click ‘Update Removal Keyframe’ to save the manual exclusions performed.
    iv. Click ‘Stop Removing Signals’ in the Manual Removal Panel to terminate the manual exclusion process.
14. Save data collected in dNEMO to mat-file and excel spreadsheet.
  a. Navigate to File > Save to output results. This will result in a number of output files which are detailed later in this protocol and within dNEMO’s documentation on Database: https://github.com/recleelab, https://doi.org/10.5281/zenodo.6841307.

## Expected outcomes

For over two decades, techniques such as single molecule fluorescent in situ hybridization (smFISH) have been powerful tools to resolve single molecules of RNA (Femino et al., 1998; Raj and van Oudenaarden, 2008). Studies using smFISH have revealed mechanisms of gene expression and numerous consequences of cell-to-cell heterogeneity but are generally limited to single timepoints because hybridization requires cell fixation. As a live-cell reporter, transfection of HeLa cells with SunRISER components enables high-intensity and photostable labeling of individual mRNA molecules that can be imaged by wide-field fluorescence microscopy for many hours (Figure 3). When analyzed with dNEMO, the number and intensity of mRNA spots can be quantified over time in each fame of the time-lapse image (Figures 3C and 3D). After the mRNA spots are detected across frames of the time-lapse images, cells can be manually segmented, automatically segmented using the Cellpose software, or a combination thereof to generate single-cell time-lapse RNA datasets. These results are written to an excel file and several mat-files, one (‘full_results’ mat-file) which can be reloaded into dNEMO for additional analysis at later time points (Figure 3D). An optional AVI file depicting circles around transcripts detected within the segmented cells as displayed in the dNEMO interface can also be created by the user by navigating to File > Save as AVI.

## Quantification and statistical analysis

Additionally included in the MATLAB version of the software package for this protocol is an interface for batch processing a folder of image files to automate spot detection and cell segmentation. To run, ensure the ‘batch_processing’ folder is both within the dNEMO directory and on the current path in MATLAB and type the following into the command window:

>RUN_ME_BATCH

On the lefthand side of the interface, select the input directory containing image data and the output directory to save results. The center panel contains the parameters for detection of spots, keyframing spots’ features, Cellpose segmentation import parameters, and additional settings. These can be interacted and set manually, or a text file containing preset parameters can be loaded into the workspace by clicking the ‘Browse’ button on the top of the center panel. At the bottom of the center panel, you can select which of the 2 major operations (dNEMO and/or Cellpose) should be propagated over the selected input image directory. The right panel of the interface contains a log window which records what operations are happening on which input files. When a valid input directory, output directory, and operation settings are selected, click ‘Confirm Valid Input Arguments’ to lock in the settings and ‘Start’ to run operations over the selected input directory. For full details on the arguments input into the batch processing tool, please see Database: https://github.com/recleelab, https://doi.org/10.5281/zenodo.6841307.

## Limitations

When adapting SunRISER for cell lines other than HeLa, the promoters used in HeLa cells may require optimization to achieve the most effective expression levels for SunRISER labeling. It was previously shown in (Guo and Lee, 2022) that SunRISER is capable of resolving small numbers of transcripts in cells, and while SunRISER is optimized for expression on the order of hundreds of transcripts, caution in interpretation of results should be observed for mRNA numbers exceeding the order of thousands per cell. We suggest using SunRISER as a starting point and further calibrate the strengths of promoters in your cell lines to reach desirable labeling. The expression ratio of protein components in different cell types may similarly need to be adjusted through the use of different promoters to reach the 5:1 protein expression ratio for optimal mRNA labeling. As with other live-cell reporter systems, SunRISER can introduce significant changes to your GOI and alter its normal function. We recommend that orthogonal techniques should be used where possible to verify the biological results obtained using SunRISER. Photobleaching of fluorescent reporters in wide-field fluorescent microscopes can vary widely between different microscope setups. In our experience with HeLa cells, 24-h imaging with 10′ intervals between frames is routinely accomplished with a DeltaVision Elite microscope. We believe with high signal-to-background, SunRISER is generally resistant to photobleaching and capable of even longer imaging experiments than described here, although this may depend on cell culture conditions and may vary between cell types. We note that we have used SunRISER successfully in human cancer cell lines and although we expect SunRISER will work in many cellular systems, extensions to primary cell lines and in vivo experiments have yet to be established. Settings for the exposure, axial spacing, and time-lapse duration may require optimization specific to other experimental designs that balance photostability, phototoxicity, as well as mRNA signal-to-background and spot detection. See (Guo and Lee, 2022) for full details on long-term imaging of mRNA molecules with SunRISER.

## Troubleshooting

### Problem 1

Decreased cell viability after SunRISER delivery.

### Potential solution

To reduce the toxicity associated with transfection, endotoxin-free plasmid can be used and the specific conditions of transfection (e.g., amount of FuGENE® HD Transfection Reagent/DNA mixture, incubation time) should be considered.

### Problem 2

High fluorescent background in cells and low signal-to-background for SunRISER-labeled mRNAs.

### Potential solution

Excessive GFP concentrations within the cells can result in a high background intensity and affect signal-to-background. Reducing the expression level of GFP could lower the basal fluorescence in transfected cells. Also check the microscope setting to find exposures that achieve the recommended intensity range. In addition, SunRISER is optimized to label mRNAs with expression levels up to several thousand transcripts per cell. GOIs expressed with higher mRNA numbers may require additional optimization. In Hela cells, cmv and ubc promoters have been shown to achieve a 5:1 ratio of protein expression. In different target cells it may be necessary to confirm that these two promoters produce the optimal expression ratio for SunRISER components.

### Problem 3

Highly variable intensity and size of mRNA spots.

### Potential solution

When SunRISER is introduced to cells via transient transfection, different cells will show significant variations depending on the amount of DNA they receive. We advise choosing cells that express SunRISER at relatively low to moderate levels to focus on cells that are not undergoing stress responses to extreme overexpression. Within one cell, we expect the intensity and size of spots are reasonably consistent and should appear as diffraction-limited spots bounded by the physical limits of the microscope. Significant variation between spot intensity within the same cell could result from the suboptimal ratio of SunRISER components, and we would generally consider this cell unsuitable for imaging or further analysis. If suboptimal labeling is frequent, we recommend switching to low passage cells and check routinely for Mycoplasma.

### Problem 4

Loss of focus during long-term imaging.

### Potential solution

Check autofocus settings and consider reducing the number of positions imaged within a single experiment. We typically do not choose regions of the cell culture well that are confluent and instead select areas where at least 20% of the region of interest is unoccupied.

### Problem 5

Spots are present in the image but dNEMO does not appear to be detecting all spots.

### Potential solution

Confirm that the axial resolution value is set appropriately. If imaging 3D stacks with a larger distance between each slice (> 1 μm) it is advisable to reduce the axial resolution to ensure most diffraction-limited spots are detected. Also check settings in dNEMO to be sure spots are not being omitted for not being detected in at least the minimum number of slices (Frame Limit, Figure 4B). The 3D image can be viewed slice-by-slice in the dNEMO interface by navigating to Image > 3D Display and selecting ‘Full 3D Stack’.

Confirm that any user-defined thresholds for spot properties are not defined too narrowly so as to omit spots from consideration (Figures 6A and 6B). User-defined spot feature keyframes can be examined and deleted in the ‘Keyframes’ panel by clicking on feature keyframes in the ‘Manual Curation’ dropdown menu (Figure 6C).

### Problem 6

Cellpose is unable to run from dNEMO (Cell Masks > Run Cellpose).

### Potential solution

Consult with the ‘READ_ME’ file in dNEMO’s documentation on Database: https://github.com/recleelab, https://doi.org/10.5281/zenodo.6841307 to ensure that the associated text file storing the location to your system’s copy of Python3 is correct. Cellpose requires Python 3 to run, and MATLAB can by default navigate to Python 2 when both Python 2 and 3 are installed on the system.

Resulting masks from Cellpose or another automated segmentation software can also be imported into dNEMO by navigating to Cell Masks > Import Cell Mask TIFF (Figure 5A).

### Problem 7

The segmentations imported from Cellpose are inaccurate and need to be altered or deleted.

### Potential solution

Segmentations imported from Cellpose can be adjusted by either clicking on ‘Modify Cell’ in the ‘Cells’ panel to modify the given segmentation or by right-clicking the cell in question from the ‘Cells’ section of the ‘Keyframes’ panel and selecting ‘Quick Seg. Redraw’ to manually re-segment the cell (Figure 5D).

The start and stop point of a tracked cell can be similarly adjusted. Right click on the cell in question in the ‘Cells’ section of the ‘Keyframes’ panel and select ‘Reset Cell Start/Stop’ to set the start and stop point of a given cell. This will delete any segmentations of the cell that lie outside the range of frames defined as the cell’s starting and stopping time point (Figure 5D).

### Problem 8

Individual objects are being detected as clusters of smaller objects (oversegmentation).

### Potential solution

If oversegmentation of valid detected spots is broadly happening, the ‘Wavelet Level’ parameter may need to be increased in the detection settings. Navigate to the Settings > Signal Parameters in the interface (Figure 4B) and increase the Wavelet Level setting to detect larger objects as single spots. Note that this will results in less reliable detection of smaller objects in the image.

There is an additional operation that is meant to reduce instances of oversegmentation in dNEMO. This operation is set on by default but may have been turned off inadvertently. Navigate to this setting in Settings > Signal Parameters and confirm that the ‘Oversegmentation Check’ setting is set to ‘Yes’.

If the image contains artifacts which are larger than diffraction-limited spots (Figure 2C) and are clearly not mRNA transcripts that dNEMO is identifying as multiple valid spots, the manual exclusion tool can be used to remove the artifacts from consideration (Figure 6C, right).

## Resource availability

### Lead contact

Further information and requests for resources and reagents should be directed to and will be fulfilled by the lead contact, Dr. Robin E. C. Lee (robinlee@pitt.edu).

### Materials availability

All reagents used have been cited in the key resources table. Plasmids generated in this study are available from Addgene (plasmid IDs: 185794–185802).

## Data Availability

Original data have been deposited to Mendeley Data: https://doi.org/10.17632/8j4x6dj2f7.1. All original code been deposited on a GitHub repository (Database: https://github.com/recleelab/, https://doi.org/10.5281/zenodo.6841307), as well as on Mendeley Data where executable files are also available (Mendeley Data: https://doi.org/10.17632/8j4x6dj2f7.1). Any additional information required to reanalyze the data reported in this paper is available from the lead contact upon request.
